# Supplementary material for: Epigenetic Signatures of Smoking in Five Brain Regions
Source: J Pers Med. 2022 Apr 2;12(4):566. doi: 10.3390/jpm12040566 (PMC9029407; doi:10.3390/jpm12040566)
Supplement: Supplementary file 1 [file jpm-12-00566-s001.zip › Supplementary_Figures.pdf]

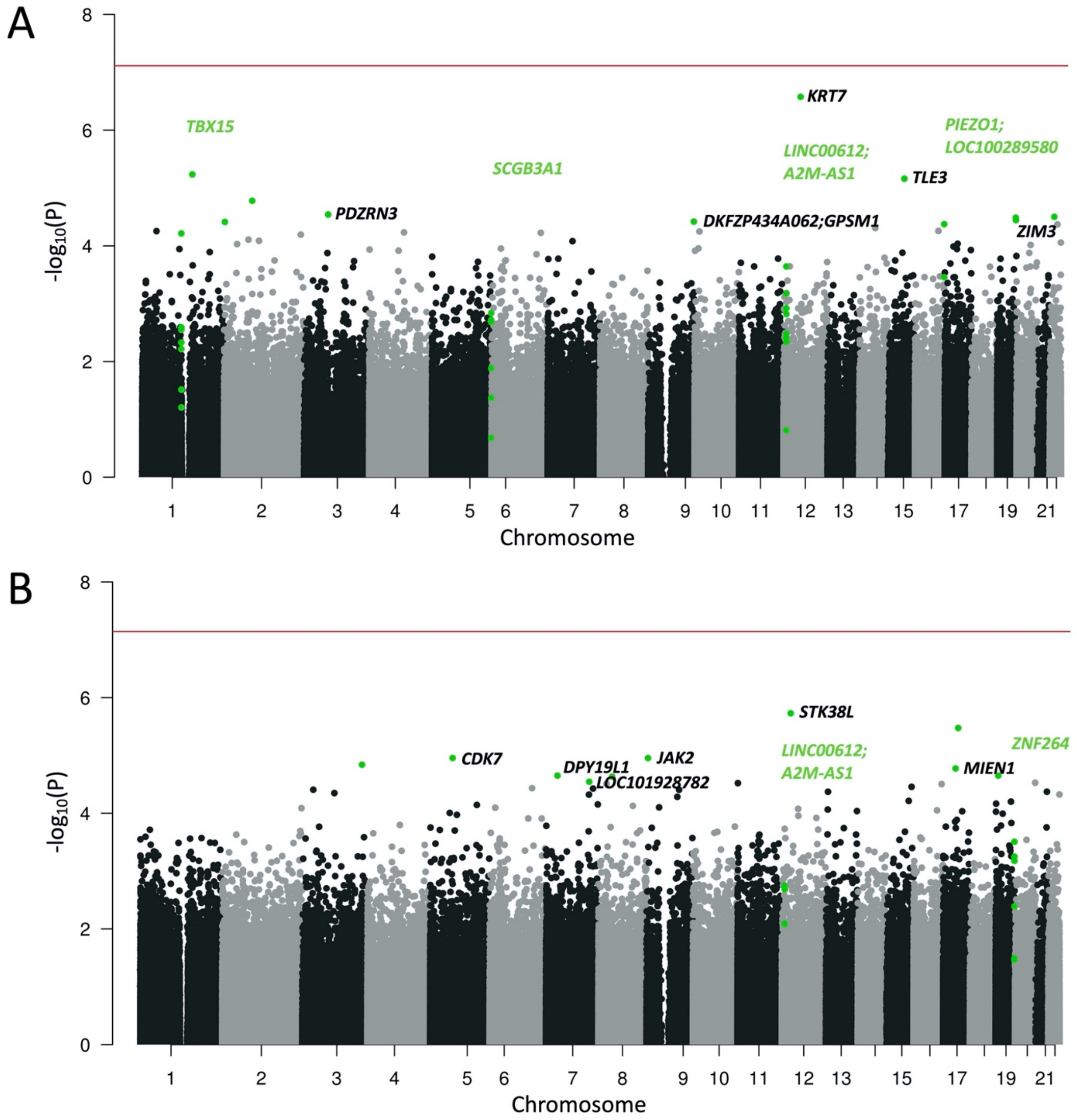

Supplementary Figure S1 | EWAS of smoking in CN and PUT

Manhattan plots for the EWAS of smoking in **(A)** caudate nucleus (CN) and **(B)** putamen (PUT). Top 10 smoking-associated CpG sites for each region and CpG sites contributing to differentially methylated regions (DMRs) are highlighted in green. Annotation to gene names was performed where possible. Names of genes harboring a DMR are highlighted in green. The horizontal line depicts epigenome-wide significance (5% FDR, for further information, see Supplementary Table S3).
