# Supplementary material for: Epigenetic Signatures of Smoking in Five Brain Regions
Source: J Pers Med. 2022 Apr 2;12(4):566. doi: 10.3390/jpm12040566 (PMC9029407; doi:10.3390/jpm12040566)
Supplement: Supplementary file 1 [file jpm-12-00566-s001.zip › Supplementary_Information.pdf]

Supplementary Information 1

Linear models used for the EWAS of smoking in different brain regions

anterior cingulate cortex (ACC) + putamen (PUT):

$$DNAm \sim smoking\ status + age + sex + PMI + neuronal\ cell\ fraction + AUD\ status + PC1_{cp} + PC2_{cp} + PC3_{cp} + PC4_{cp} + PC5_{cp} + PC6_{cp} + PC7_{cp} + PC8_{cp} + PC9_{cp} + PC10_{cp}$$

Brodmann Area 9 (BA9):

$PC1_{cp}$ and $PC7_{cp}$ removed due to multicollinearity

$$DNAm \sim smoking\ status + age + sex + PMI + neuronal\ cell\ fraction + AUD\ status + batch + PC2_{cp} + PC3_{cp} + PC4_{cp} + PC5_{cp} + PC6_{cp} + PC8_{cp} + PC9_{cp} + PC10_{cp} + PC1_{geno} + PC2_{geno} + PC3_{geno} + PC4_{geno}$$

caudate nucleus (CN) + ventral striatum (VS):

$PC5_{cp}$ removed due to multicollinearity

$$DNAm \sim smoking\ status + age + sex + PMI + neuronal\ cell\ fraction + AUD\ status + batch + PC1_{cp} + PC2_{cp} + PC3_{cp} + PC4_{cp} + PC6_{cp} + PC7_{cp} + PC8_{cp} + PC9_{cp} + PC10_{cp}$$
